# The association between C-reactive protein and the likelihood of progression to joint replacement in people with rheumatoid arthritis: a retrospective observational study

**DOI:** 10.1186/1471-2474-9-146

**Published:** 2008-11-04

**Authors:** Chris D Poole, Pete Conway, Alan Reynolds, Craig J Currie

**Affiliations:** 1 360° Research Limited, Penarth, UK; 2 Health Economics, Wyeth Europa Limited, Maidenhead, UK; 3 Scientific Affairs, Wyeth Pharmaceuticals, Maidenhead, UK; 4 Department of Medicine, School of Medicine, Cardiff University, Cardiff UK; 5 Health Evaluation Associates, The Pharma Research Centre, Cardiff MediCentre, Cardiff CF14 4UJ, UK

## Abstract

**Background:**

This study sought to evaluate the association between systemic inflammation as measured by C-reactive protein and total joint replacement and the association between change in CRP status (low, ≤ 10 mg/L and high, >10 mg/L) measured over one year and total joint replacement in patients diagnosed with rheumatoid arthritis.

**Methods:**

A cohort of patients was selected from The Health Improvement Network (THIN) dataset of anonymised patient-level data from UK general practice with a confirmed chronic rheumatic diagnosis. Surgery-free survival was evaluated using Cox proportional hazards regression models (CPHM).

**Results:**

2,421 cases had at least one CRP measurement of which 125 cases (5.2%) had at least one major joint replacement. In CPHM, each additional unit increase in log mean CRP (range 1 to 6) was associated with a hazard ratio (HR) for major orthopaedic surgery of 1.36 (95% CI 1.10 to 1.67; p = 0.004), after controlling for age at first rheumatoid presentation and average body mass index over the same observation period. Repeated CRP observations around one year apart were recorded in 1,314 subjects. After controlling for confounding factors, in cases whose CRP remained high (>10 mg/L), the HR for joint replacement increased more than two-fold (p = 0.040) relative to cases whose CRP remained low. In patients whose CRP increased from low to high, the HR was 1.86 compared to those who remained in a low state (p = 0.217). By comparison, among those subjects whose CRP was reduced from a high to low state, the hazard ratio was more than halved (1.46) from to those who remained high (p = 0.441). Although underpowered, the trend evident from CRP change corroborates the association of TJR progression with mean CRP.

**Conclusion:**

CRP level predicts progression to major joint replacement after standardisation for relevant risk factors as did change in CRP status between low and high states observed over one year.

## Background

Rheumatoid arthritis (RA) is a chronic illness characterised by inflammation of the synovial tissue in joints which can lead to joint destruction. Despite early and more aggressive treatment policies aimed at limiting the disease pathology rather than just controlling symptoms, progressive destruction of joints continues in a subgroup of patients who eventually require surgery. The ERAS Group inception cohort provides the most accurate data for the UK; in the first five years of observation seventeen percent of RA patients treated with conventional therapy required orthopaedic surgical intervention, 41% of whom underwent major joint replacement [1]. This is consistent with other observational studies [2], which also showed that by 10 years 20% of RA patients require surgery [3], and within 20 years a quarter of all patients will have undergone total joint arthroplasty (TJA) [4].

Although a significant long-term outcome in RA, joint replacement surgery is currently excluded from accepted economic models evaluating the cost-effectiveness of disease modifying anti-rheumatic drugs (DMARD) because of the absence of data on the effects of DMARDs on joint replacement rates [5]. The long-term impact of tumour necrosis factor alpha (TNF-α) inhibitors on joint failure and the likelihood of orthopaedic surgery cannot be demonstrated directly at present because the agents are still relatively new. Surrogate end-points such as radiographic change suggest potentially important benefits [6], and potentially a reduced demand for surgery, but the clinical relevance of reported radiographic changes is debated [7].

Surrogate markers of inflammation measured by laboratory assay such as erythrocyte sedimentation rate (ESR) and C-reactive protein are central in the evaluation of disease progression and response to therapeutic intervention [8-10]. Although the baseline prognostic value of these inflammatory markers for joint replacement has been assessed [1,11], few studies have looked at their predictive value when assessed over the lifetime of the disease. Furthermore no study appears to have examined the relationship between longitudinal change in CRP and orthopaedic prognosis, as has been true for evaluation of the effect of such changes upon the risk of cardiovascular disease in other patient groups [12].

In this study, we sought to examine effect of both average CRP and CRP change on progression to total joint replacement using routine laboratory data recorded in general practice among patients diagnosed with rheumatoid arthritis.

## Methods

Data analysed in this study were derived from routine general practice in the UK. These data were sourced from a proprietary health data resource, The Health Improvement Network (THIN) [13], a competitor to the General Practice Research Database (GPRD) [14], a widely recognised source of UK GP data. THIN was selected since it had greater patient numbers matching the initial selection criteria.

THIN data are collected in a non-interventional way from the daily record keeping of primary care physicians in the UK. The records are anonymised at the collection stage so that researchers have access to an encrypted identifier for the physician's office and the patient. They provide a longitudinal medical record for each patient. Currently, the dataset consists of contributions from over 300 practices and data from approximately five million patients of whom over 2.3 million are actively registered with the practices and can be prospectively followed. The remaining patients have historical data but have either left the practice or died. There are nearly 30 million patient years of computerised data in THIN. On average, patients have full data for six years and may have up to 15 years of observations. Data from THIN consist of four categories that detail the following: 1) subject demographic details; 2) medical history (diagnoses); 3) test results and addition health-related data such as smoking status; and 4) drug treatments. Ethical approval was granted by the Cambridge MREC.

### Patient selection

Data were extracted for patients with a diagnosis for rheumatoid arthritis (RA) recorded at least twice on two different dates. A marker of 'newly diagnosed' was assigned if the patient had less than six months case history prior to first RA diagnostic code.

Of these patients, the first rheumatoid presentation (index event) was determined from the first of the following three events: either 1) occurrence of three consecutive monthly prescriptions for a non-steroidal anti-inflammatory drug (NSAID); or 2) recorded rheumatology referral, consultation or screening; or 3) an RA diagnosis. Cases were further selected by presence of at least one valid CRP measurement between the index date (the date of the index event) and study endpoint.

Finally, to be assured of following cases from their first rheumatoid presentation, a minimum wash-in period of six months between the date of first contact with the database and the index date was applied.

### Endpoint determination

The observational endpoint was the first recorded date of a major orthopaedic intervention which included hip, knee, shoulder, elbow joint replacement or cervical spine fusion, in common with the Early Rheumatoid Arthritis Study Group [1]. Those who did not progress to TJR were censored at the date of last contact or death.

### Laboratory data

The key marker in this investigation, serum CRP concentration, is reported as provided in THIN. CRP observations were utilised only if they had a value >0 in order to be sure that this was a true observation and not a null value attributed 0 (zero) systematically or at data entry. Recorded total cholesterol (TC) observations were similarly treated. Mean CRP was calculated for all valid observations between index and study endpoint, as was mean body mass index (BMI) and mean blood pressure (systolic and diastolic).

Cases where CRP change was recorded were included where at least two observations were recorded approximately one year apart (± 90 days in order to recruit sufficient cases). Given high intra-individual variability for CRP [15,16], baseline CRP status was calculated as the average of all observations within 90 days of the first CRP observation. Similarly, the follow-up CRP level was defined as the mean of values occurring within a further 90 day of the second (1 year) observation. It was not possible to determine whether the assays were of high sensitivity therefore decided to categorize CRP level according to the scientific statement recently issued by the American Heart Association and Centers for Disease Control and Prevention [17] a method used previously in large epidemiological studies [18]. A low level of CRP was defined as 10 mg/L or less whilst levels in excess of 10 mg/L were defined as high based on published population norms. CRP change was then defined for the period as low → low (LL), low → high (LH), high → low (HL), and high → high (HH). Classification by the method used in a recent study of hospital laboratory data [19] was not feasible in this instance as too few cases in the CRP change subgroup met the AHA criteria for 'normal' CRP (≤ 3 mg/L). Any case in the CRP change subgroup who had a recorded diagnosis of an acute ischaemic event during their CRP observation period (± 30 days) was excluded from analysis.

### Statistical methods

Survival was evaluated using Cox proportional hazards regression analysis (CPHM), using SPSS^® ^v15, SPSS Inc, Chicago. Independent covariates of survival were tested using a manual forward inclusion method with a threshold significance of p = 0.05 as the criterion for inclusion in the final models. Time to event was measured from the date of the last reported CRP observation. Surviving cases were right-censored by their last known contact date.

## Results

Among 7,121 cases with 'newly-diagnosed' rheumatoid arthritis, 3,576 cases had at least one valid CRP measurement of which 2,421 (34% of the initial sample) had a 6-month run-in prior to their first rheumatoid presentation. There were 125 major joint replacements (5.2%) in this cohort, the majority (95.2%) being of the hip or knee (39.2% and 56.0% respectively). The median time from RA diagnosis to TJR was 49 months (IQR: 25 to 104). In total there were 24,023 CRP observations from the 2,421 cases, 76% of whom had more than one CRP measurement. The median number of CRP observations per case was 4 (IQR: 2 to 13) and the median time to event (TJR or censor) was 117 months (IQR: 71 to 161). There was no statistically significant difference in the frequency of CRP testing between those who progressed to TJR and censored cases (p = 0.122).

Cases progressing to TJR were significantly older at first presentation than non-TJR cases (59.5 vs. 51.4 years; p < 0.001; had a higher average BMI prior to major surgery (28.1 kg/m^2 ^vs. 26.9 kg/m^2^; p = 0.051); had higher average systolic blood pressure (145 mmHg vs. 137 mmHg; p < 0.001); and had higher mean CRP prior to surgery (19.16 mmol/L vs. 12 mmol/L; p < 0.001; Table 1).

**Table 1:** Subject characteristics by study outcome

	**All subjects**		**No joint replacement**		**TJR**		***p***
number (%)	2,421		2,296	94.4%	125	5.2%	
% male	26.0%		26.0%		26.4%		0.917
Mean age at index (SD)	51.9	15.0	51.4	15.0	59.4	11.5	<0.000
Prior ischaemic disease (%)	10.1		10.0		10.4		0.878
Median GP visits yr prior (IQR)	10	(6–18)	10	(6–18)	10.5	(6–18)	0.781
Mean BMI before CRP (SD)	26.9	5.6	26.9	5.6	28.1	6.3	0.051
Mean SBP before CRP (SD)	137.4	16.6	137.0	16.5	145.1	16.1	<0.000
Mean TC before CRP (SD)	5.35	1.00	5.35	1.00	5.45	0.93	0.388
Ever smoked (%)	38.4		38.8		31.5		0.107
Ln mean CRP (SD)	2.51	1.01	2.49	1.00	2.95	0.98	<0.000
Number of deaths (%)	194	8.0	185	8.1	9	7.2	0.866

Index – 1^st ^rheumatoid event; TJR – major orthopaedic intervention (ERAS definition) – standard deviation; IQR – inter-quartile range;

BMI – body mass index (kg/m^2^); SBP – systolic blood pressure (mmHg); TC – total serum cholesterol (mmol/L); Ln – natural logarithm.

Cases from the initial sample with no recorded CRP results were an historically older cohort. Their median year of first RA diagnosis was 1997 (IQR: 1993 to 2001) whilst for those with CRP measurements median year of diagnosis was 2001 (IQR: 1997 to 2003, p < 0.001).

### Mean CRP measurement

**Figure 1:**
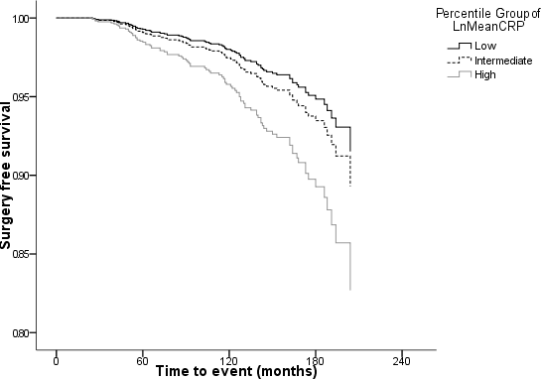
Surgery-free survival by mean CRP thirtile (low [<8.03 mmol/L], intermediate [8.03 to 18.32 mmol/L], and high [>18.32 mmol/L]) standardised for age, and average BMI.

CPHM showed each unit increase in log mean CRP was associated with a 36% increase in the HR for TJR (95% CI 10% to 67%; Table 2) after controlling for age at index, and mean BMI. Gender, mean total cholesterol, mean SBP, smoking status, prior cardiovascular morbidity, and number of visits to the GP in the year prior to CRP measurement were not significant covariates in the presence of those factors remaining in the model. Survival curves for each thirtile of mean CRP (adjusted for index age and mean BMI) are shown (Figure 1).

**Table 2:** Cox proportional hazards model for first total joint replacement (n = 1,885; 3.6% events)

**Variables in the Equation**	**β**	**SE**	***p***	**HR**	**95% CI for HR**	
					*Lower*	*Upper*
Age at index	0.044	0.009	0.000	1.04	1.03	1.06
Ln mean CRP	0.307	0.106	0.004	1.36	1.10	1.67
Mean BMI	0.041	0.018	0.022	1.04	1.01	1.08

Covariates not reaching threshold significance: gender; mean total cholesterol; mean BP; smoking history; cardiovascular morbidity; prior year GP attendance.

β – parameter estimate; SE – standard error of β;*p *– statistical significance; HR – hazard ratio;

CI – confidence interval.

### CRP change

Repeated CRP observations at one year were available for 1,287 subjects, of whom 54 experienced at least one major joint replacement. The median number of tests in the quarter following first-ever CRP observation was 1 (IQR: 1 to 2). In the follow-up period (9 to 15 months after first CRP) the median number of CRP observations was 2 (IQR: 1 to 4).

**Figure 2:**
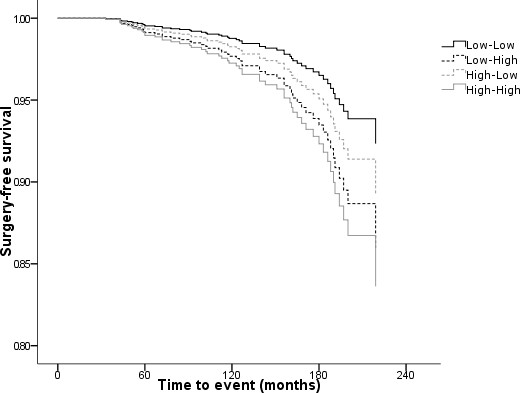
Surgery-free survival by 1-year CRP change status standardised for age (CRP status: Low, ≤ 10 mmol/L; High, >10 mmol/L).

In a CPHM model of all-cause mortality controlling for age at index, the hazard of joint replacement showed a trend between CRP change categories consistent with the finding for mean CRP (Table 3 & Figure 2). Compared to stable low-CRP cases, the hazard ratio of TJR among persistently high-CRP was approximately doubled (HR 2.19 [95%CI 1.04 to 4.64; p = 0.040]). Among those whose CRP increased from low to high, the hazard ratio was 1.86 (95%CI 0.69 to 5.01) but this observation did not reach threshold significance (p = 0.217). The hazard ratio for those whose CRP returned from an elevated to a low state, showed no statistically significant difference from the stable low-CRP reference group (HR 1.46 [95%CI 0.56 to 3.78; p = 0.441]).

**Table 3:** Cox proportional hazards model for first total joint replacement after one-year CRP observation (n = 1,230; 4.2% events)

**Variables in the Equation**	**B**	**SE**	***p***	**HR**	**95% CI for HR**	
					*Lower*	*Upper*
Age at index	0.061	0.012	0.000	1.06	1.04	1.09
1-year CRP change (*cf. Low-Low*)			0.203			
Low to High	0.623	0.504	0.217	1.86	0.69	5.01
High to Low	0.375	0.487	0.441	1.46	0.56	3.78
High-High	0.786	0.382	0.040	2.19	1.04	4.64

Covariates not reaching threshold significance: gender; time to 1st CRP observation.

Low CRP defined as ≤ 10 mmol/L; High CRP defined as >10 mmol/L

The time dependency of CRP was tested to ensure compliance with CPHM assumptions. First CRP was not correlated with time from index (r_Pearson _= -0.032, p = 0.121) and mean CRP was not correlated with time from first CRP to last CRP (r_Pearson _= -0.042, p = 0.072).

## Discussion

This is the first study to demonstrate that increased mean CRP observed throughout the disease process is associated with accelerated progression to total joint replacement for patients with rheumatoid arthritis. Further, the change in serum CRP between low and high levels also appears correlated with progression to major surgical endpoints, suggesting that the risk is modifiable. An absence of statistical significance in these last findings would have been expected from the diminished number of events in this reduced cohort. The concordance of results using both a statistical classification (thirtiles of mean CRP) and an a priori population-based classification (for CRP change) serves to strengthen the findings.

In this cohort the median time from diagnosis to joint replacement was 49 months, correspondent to the upper end of centres contributing to the UK-based ERAS study [1] in which the overall median time from baseline was 36 months. This figure does differ from other reported estimates which put median time at 7.0 [20] years (US-based outpatient study) for any total joint arthroplasty and around 14 years for total replacement of large joints (Finnish outpatient study) [21]. It is likely in this primary care dataset that case ascertainment is underestimated. The current data span a 20 year period in which significant changes in the management of RA has occurred which would reasonably have been expected to modify practice primary care. The ERAS study represents an assessment of relatively recent rheumatology best practice. Thus, hazard ratios are likely to be reliable; however absolute risk is likely to be underestimated because of the probability of under-reporting by the GPs. This under-reporting is unlikely to be systematic. These purely observational primary care cases will incorporate additional delays such as referral and patient non-compliance. A further source of underestimation comes from our inclusion of non-survivors in the censored cohort. A decision was taken to include these cases to provide a more relevant estimate including other outcomes from RA, including mortality.

Despite their limitations, these findings do illustrate the association between mean CRP change over the course of the disease process and subsequent need for joint replacement. As such CRP could reasonably be used as a surrogate marker in modelling endpoints for the comparison of disease modifying anti-rheumatic drug treatments where no long-term outcome data exists. Given that currently accepted cost-effectiveness models for DMARDs do not include joint replacement as an endpoint gives added weight to the utility of these findings. An analogous situation would be the exclusion of microvascular and macrovascular endpoints from the current economic modelling of new hypoglycaemic agents. In common with the TNF-α blockers, neither the clinical trials programme nor extensive post-marketing surveillance for hypoglycaemic treatment could reasonably be expected to show a direct reduction in these long-term endpoints yet improvement in HbA1c, is universally accepted a surrogate for disease control [22].

As a marker of disease status, CRP has limitations. CRP levels change more readily in response to other inflammatory stimuli such as minor infections and surgery. Erythrocyte sedimentation rate (ESR) could have provided a more stable surrogate for disease status but results were not sufficiently available in this register study. Counts of swollen and tender joints presented during follow-up would also have provided a more accurate picture of disease status but are poorly recorded in primary care data and may have been difficult to quantify over time. Other direct assays of type I collagen destruction such as ICTP have shown good prognostic ability for TJR [23] but are not routinely recorded.

The current observation that change in CRP modifies the risk of joint progression will also be of interest to those assessing disease prognosis in rheumatoid disease. As this study could only demonstrate association and not causation, further studies will consider which therapeutic interventions control inflammation most effectively, and whether direct benefit in limiting progression to joint replacement can be characterised.

## Conclusion

Therapeutic practice is evolving in rheumatoid arthritis with the introduction of biologic agents which provide rapid, profound and sustained suppression of disease activity in correspondence with a marked reduction in CRP levels [24]. In 2007, primary care prescriptions for adalimumab and etanercept accounted for 0.4% of all DMARDs [25], making their current presence on practice databases vanishingly small. Their long-term benefit on relevant endpoints such as TJA cannot be established until a substantial body of practice data has accrued. Until then surrogates of the disease process including inflammatory markers can give an indication of the likely prognosis when these agents are used. The present analysis suggests that one of these surrogates, CRP, has value in the prognosis of TJA.

## Competing interests

CP and CJC were privately funded by Wyeth Europa Ltd. PC and AR are employees of Wyeth Europa Ltd. The THIN study data was acquired under the terms of a subscription to THIN held by Wyeth Pharmaceuticals.

The study received ethical approval from the Cambridge MREC (reference number 08/H0305/46).

## Authors' contributions

AR conceived the study idea. The study was designed jointly by all four authors. CP carried out most of the initial data analysis along with CJC. CP wrote the first draft and the remaining three authors edited earlier versions and agreed to the final draft. CP & CJC jointly addressed the revisions.

## Pre-publication history

The pre-publication history for this paper can be accessed here:


